# A Novel Mutation in an NPXY Motif of β Integrin Reveals Phenotypes Similar to *him-4/hemicentin*

**DOI:** 10.3389/fcell.2019.00247

**Published:** 2019-10-28

**Authors:** Zhongqiang Qiu, Peter Sheesley, Jeong H. Ahn, Eun-Jeong Yu, Myeongwoo Lee

**Affiliations:** Department of Biology, Baylor University, Waco, TX, United States

**Keywords:** *pat-3*, basement membrane, negatively charged, glutamic acid, germ cell, male mating, somatic gonad, migration

## Abstract

Integrin, an αβ heterodimeric cell surface receptor for the extracellular matrix (ECM), carries two tyrosine phosphorylation motifs in the cytoplasmic tail of the β subunit. NPXY (Asn-Pro-x-Tyr) is a conserved tyrosine phosphorylation motif that binds to the phospho-tyrosine binding (PTB) domain. We generated a tyrosine to glutamic acid (E) mutation to modify tyrosine (Y) into a negatively charged amino NPXY in the β*pat-3* integrin of *Caenorhabditis elegans*. The transgenic rescue animal displayed defects in gonad migration and tail morphology. Also, the mutant animals produced a high number of males, suggesting that the Y to E mutation in β*pat-3* integrin causes a phenotype similar to that of Him mutant. Further analyses revealed that males of *pat-3(Y804E)* and *him-4/*hemicentin share additional phenotypes such as abnormal gonad and unsuccessful mating. A *pat-3* transgenic rescue mutant with a non-polar phenylalanine (F) in NPXY, *pat-3(Y792/804F)*, suppressed the high male number, defective mating, inviable zygote, and the abnormal gonad of *him-4* mutants, indicating that Y to F mutation in both NPXY motifs suppressed the *him-4* phenotypes. This finding supports the idea that the ECM determines the activation state in integrin NPXY motifs; *him-4/*hemicentin may directly or indirectly interact with integrins and maintain the NPXY non-charged. Our findings provide new insight into a suppressive role of an ECM molecule in integrin NPXY phosphorylation.

## Introduction

Integrin is a cell surface receptor for the extracellular matrix (ECM), playing a significant role in cell adhesion and tissue organization. Upon binding to the ECM, integrin links ECM components to cell signaling and cytoskeletal molecules via its cytoplasmic tail. Notably, the β integrin cytoplasmic tail contains conserved tyrosine phosphorylation sites. These are NPXY (tyrosine phosphorylation) motifs essential for recruitment of focal adhesion proteins such as talin (Oxley et al., 2008) and kindlins (Larjava et al., 2008; Moser et al., 2009; Plow et al., 2009). This interaction facilitates the connection of integrins to the actin cytoskeleton as well as activating integrin signaling.

**FIGURE 1:**
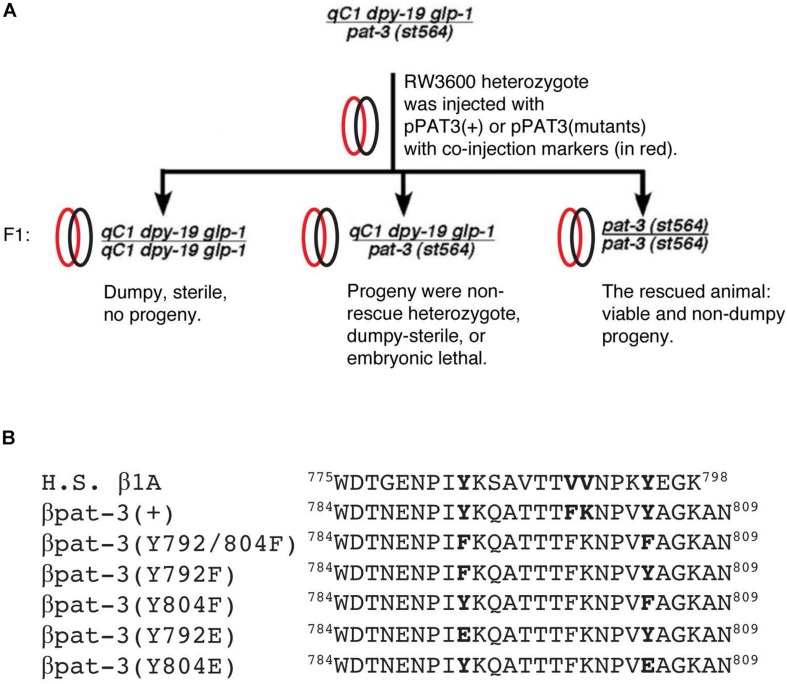
Strategy to rescue *pat-3(st564)* and β integrin cytoplasmic tails. **(A)** *pat-3* null rescue scheme; **(B)** cytoplasmic tails from *Homo sapiens* (H. S.) β1A integrin and *C. elegans* β *pat-3* were compared. For mutant β*pat-3*, one or both tyrosine (Y) were mutated to phenylalanine (F) or glutamic acid (E). Protein sequences from downstream of a unique tryptophan (W^784^) are aligned.

Integrin is an excellent molecule to study the function of the NPXY motif, because two NPXY motifs are conserved in many species and arranged in the cytoplasmic tail of β integrins (Calderwood et al., 2002, 2003; Calderwood, 2004) (Figure 1B). For example, mutations in NPXY motifs in β3 integrin are linked to mice with a tendency to rebleed after clotting (Law et al., 1999). Additionally, the collecting duct cells expressing β1 integrin with Y to phenylalanine (F) mutation showed failed tubulogenesis in 3D collagen culture (Mathew et al., 2012). Many studies

have examined the function of NPXY in the β integrin cytoplasmic tail and found that the Y to F mutation sustains integrin functions while the mutation to alanine (A) abolishes the cellular function of β integrin (Czuchra et al., 2006), suggesting the indispensable nature of the Y or a similar amino acid for the function of β integrin.

*Caenorhabditis elegans* is an excellent genetic model organism to study integrin function, having two α and one β chains (Kramer, 2005). *pat-3*β integrin (PAT-3) is expressed in muscles, gonads, and neurons and plays essential roles in tissue organization (Gettner et al., 1995; Ono et al., 2007). Null mutations in β *pat-3* integrin display embryonic lethality (Williams and Waterston, 1994), while dominant negative PAT-3 tail (HA-βtail) revealed that *pat-3* is essential for muscle filament organization, gonad migration and ovulation (Lee et al., 2001). RNAi of *pat-3* parallels the dominant negative PAT-3 tail (HA-βtail) phenotypes. This demonstrated that the role of integrin in postembryonic tissues justifies further analyses of β integrin cytoplasmic tail (Lee et al., 2005).

There have been several reports about the NPXY motif in *pat-3*. For example, Y to F mutations in β*pat-3* integrin caused gonad migration defects in hermaphrodites (Xu et al., 2010). The DEP-1, R3 receptor phospho-tyrosine phosphatase, dephosphorylates the membrane proxy NPIY^792^ and plays a vital role as a negative regulator of vulva formation (Walser et al., 2017), suggesting that the dephosphorylation of the membrane proxy NPXY motif is crucial for maintaining integrin function. In an effort to study the function of NPXY motifs, we have generated a mutant with the membrane distal tyrosine (Y^804^) is replaced by a negatively charged/acidic amino acid, glutamic acid (E). Although the charged E side chain appears different from the phospho-tyrosine, the Y to E mutation has been used as an analogous substitution for the phospho-tyrosine (Anthis et al., 2009; Subramanyam et al., 2016). Reszka et al. (1992) reported that the membrane proxy Y883E mutation in β_1__*c*_ avian integrin severely impaired its localization to focal adhesion and talin binding (Reszka et al., 1992). In our study, the membrane distal *pat-3(Y804E)* mutation rescued embryonic lethality of *pat-3* null and created viable transgenic animals. However, it also displayed Him (high incidence of males) and defective male mating, which is similar to that of *him-4* mutant alleles.

Vogel and Hedgecock (2001) reported that mutations in *him-4*, encoding a large ECM protein similar to human hemicentin I, cause defective cytokinesis of mitotic germ cells and consequently lead to chromosomal losses as well as production of abnormal males with a mating defect (Vogel and Hedgecock, 2001; Xu et al., 2007). Another study reported that HIM-4/hemicentin is essential in adjoining basement membranes (BM) between two adjacent tissues and that PAT-3 integrin is required for the formation of HIM-4/hemicentin puncta at the anchor cell invasion foci during vulva induction (Morrissey et al., 2014). Our genetic analysis revealed that the non-polar replacement mutant, *pat-3(Y792/804F)*, suppressed Him, abnormal gonad, and unsuccessful mating of *him-4/hemicentin* mutants, suggesting that HIM-4/hemicentin interacts with *pat-3* integrin and modulates the charged state of the NPXY. This suggests that NPXY motifs in β integrin play a vital role in its function, which impacts many aspects of development and interaction between cells and their surroundings.

## Materials and Methods

### Strains and Nematode Culture

Wild-type Bristol strain, N2, and mutant strains were purchased from the *Caenorhabditis* Genetics Center at the University of Minnesota, Minneapolis, MN. Mutant lines used in this study include *RW3600 qC1 pat-3(st564)/dpy-19(e1259) glp-1(q339) III, raIs8[unc-112:GFP* + *rol-5(su1006)]* (Rogalski et al., 2000), *lon-2(e678) him-4(e1267) X, and him-4(rh319) X.* Transgenic *pat-3* rescue animals used in this study include *mwEx443[pat-3* (+)*], mwEx35[pat-3 (Y792F)]*, *mwEx31[pat-3 (Y804F)]*, *mwEx32[pat-3 (Y792/804F)]*, and *kqEx804[pat-3(Y804E)]* (this study), which are described in Lee et al. (2001). All strains were grown on OP50 seeded NGM plates and maintained at room temperature as described.

### Nematode Genetics and Phenotype Analysis

All *C. elegans* strains were cultured on NGM plates seeded with OP50 (Brenner, 1974). Four double mutants, *mwEx35[pat-3 (Y792F)]; him-4(e1267)*, *mwEx31[pat-3 (Y804F)]; him-4(e1267)*, and *mwEx32[pat-3 (Y792/804F)]; him-4(e1267)*, were generated by crossing homozygote or heterozygote males from each transgenic line to *lon-2(e678) him-4(e1267)* (Hodgkin et al., 1979) hermaphrodites. From the F2 generation, Lon-Him worms that were 100% green (F3) were selected for further studies. For DTC defects, worms were anesthetized with 5 mM levamisole solution (in M9) on 3% agarose pads. Distal arms with abnormal trajectories such as no dorsal turns, looping back, and irregular turns were scored as gonad migration abnormal (Lee et al., 2001). For Him phenotypes, worms were self-fertilized, and males were randomly identified from agar plates. The abnormal hermaphrodite tail was scored for protrusions in the posterior end of the body and irregular folding of tail shafts. Male gonads were also scored for irregular turns. Retracted fans, fused rays, and protruded spicules were scored for male tail abnormal. For the mating success assay, three to six males and one hermaphrodite were placed on an NGM agar plate. An increased number of non-Unc in F1 progeny was a criterion for mating success. A minimum of five mating plates (except controls) per strain were set up; an increased number of males in F1 in a mating plate was scored as success (+). For *him-4* RNAi, *raIs8[unc-112:GFP* + *rol-6(su1006)]* animals were fed with *him-4* RNAi bacteria, a clone F15G9.4 (X-4F24) from the *C. elegans* RNAi library (Source Bioscience, Nottingham, United Kingdom). The localization of UNC-112:GFP was observed 72 h after feeding.

### Generation of *pat-3(Y804E)* Construct and Microinjection

To generate the *pat-3(Y804E)* mutation construct, an overlap extension method to create mutations in the ^787^NENPIY^792^ or ^799^FKNPVY^804^ sequence was performed using pPAT3-PB12K, wild-type *pat-3* gene, as a template. The pPAT3-pPB12K genomic DNA, containing 11799 bases of *pat-3* gene, was cloned between *Pvu*II and *Pst*I in pSP73 (Promega, Madison, WI, United States), described in the previous study (Lee et al., 2001). Primers were designed to make a mutation (TAC to GAG) from tyrosine (Y) to glutamic acid (E) as follows:

PAT3 Y2EFW3: 5′- CCA GTA **GAG** GCT GGA AAA GCC -3′
PAT3 Y2ERV3: 5′- GGC TTT TCC AGC **CTC** TAC TGG -3′
PAT3 EJ Y1EFW1: 5′- CCC AAT C**GA G**AA ACA GGC CAC G -3′
PAT3 EJ Y1ERV2: 5′- CGT GGC CTG TTT **CTC** GAT TGG G -3′
PAT3 FORWARD(FW)3: 5′- TGC TAG CCC TCC ACC TGT CCC TGT G -3′
PAT3 REVERSE(RV)5: 5′- CCA CAC GGT AAA TAG G -3′

To perform overlap extension, two polymerase chain reactions (PCR), PAT3FW3 - Y2ERV3 (pair 1) and Y2EFW3 – PAT3RV5 (pair 2), were prepared. Two amplified resultant DNA molecules were then mixed for the second round PCR as overlap templates for PAT3FW3 and PAT3RV5 primers. The second round DNA was digested with *Eco*RI and cloned into pSP73 vector for sequencing to confirm mutations. The sequence confirmed DNA construct was digested with *Msc*I and *Eco*RI, and the fragment was inserted back into pPAT3-PB12K (Lee et al., 2001); completed circular DNA constructs were pPAT3(Y792E) and pPAT3(Y804E). For micro-injections, 50 mg/ml of pPAT-3(Y792E) or pPAT3(Y804E), was mixed with 50 mg/ml of TG96 *sur-5:GFP* DNA (Gu et al., 1998) in TE buffer (pH = 7.5, 50 mM Tris-HCl, 1mM EDTA). The constructs were microinjected into RW3600 *pat-3(st564)/*qC1 *dpy-19(e1259) glp-1(q339)* III strain (Williams and Waterston, 1994) and selected for green and non-DpySterile segregating F1 progeny. Rescued transgenic animals usually appear in F2 animals, which produce viable, non-Dpy, carrying PAT-3(Y804E) and TG96 *sur-5*:GFP construct. Rescued animals segregate non-Dpy green transgenic and non-green Pat because they are viable only when it carries *sur-5*:GFP and *pat-3* construct (Figure 1A). The rescued *pat-3(Y804E)* was used for further studies. Alternatively, 50 mg/ml of pPAT3(Y792E) or pPAT3(Y804E) DNA was also microinjected into N2 animals to measure the dominant effect of the transgenes. Green F2 animals were isolated and were studied for analysis.

### Muscle Staining, and Fluorescence/DIC Microscopy

To examine the muscle cytoskeleton, animals were collected and placed on poly-L-lysine coated slides and fixed with methanol and acetone for 5 min each at −20°C. Fixed worms were treated with rhodamine-conjugated phalloidin (0.2 U/ml, Sigma Chem. Co.) for 2 to 3 h at room temperature. Washed samples were mounted on the Nikon TE2000-U epifluorescence microscopes. Images were captured using a *CoolSnap* ES (Roper Scientific, Tucson, AZ, United States) and analyzed with *Metavue* software (version 7.5, Molecular Devices, Downingtown, PA, United States) or *NIS Elements* (version 5.02, Nikon Instruments, Melville, NY, United States). For DIC or fluorescence live samples, animals were fed with 2% NaN_3_ or 5 mM levamisole solution on agarose pads and mounted on the Nikon TE2000-U or Eclipse Ni-U microscope. Samples were examined and analyzed for defects using a 20X or 40X Plan Fluor objective lens. Images were obtained using *Coolsnap* ES or DYNO monochrome camera (Photometrics, Tucson, AZ, United States) and analyzed using *Metavue* (version 7.5, Molecular Devices, Downingtown, PA, United States) or NIS Elements (version 5.02, Nikon Instruments, Melville, NY, United States) software.

For DNA staining, animals were washed off the plate and fixed with methanol in −20°C. The next day fixed worms were treated with ethanol series from 95–30%. After the final alcohol treatment, isolated animals were treated with 0.1 μg/ml of DAPI (4′,6-Diamidine-2′-phenylindole dihydrochloride, Sigma-Aldrich, St. Louis, MO, United States) in M9 buffer for more than 4 h with rotation. After three washes, samples were observed under the microscope.

Animals were stained with MH25, anti-PAT-3 antibodies (1:250 dilution, purchased from DSHB, Iowa City, IA, United States) (Francis and Waterston, 1991), and goat anti-mouse Cy3-conjugated antibody as secondary antibodies (1:500 dilution, Jackson Laboratory, Bar Harbor, ME, United States). Briefly, animals were placed on poly-L-lysine coated slides (Sigma-Aldrich, St. Louis, MO, United States) and fixed with ice-cold 100% methanol for 5–10 min. Fixed animals were blocked with 5% goat sera and treated with primary and secondary antibodies. Fluorescence images were obtained by using an Olympus FLUOVIEW FV1000 confocal microscope equipped with a PlanApo 60X oil-immersion objective lens and processed using its accompanying FLUOVIEW (version 4.2, Olympus Corporation, Center Valley, PA, United States) software.

Statistical analysis in Tables 1–3 and Supplementary Table S1 were performed with JMP Pro (version 14.0.0., SAS Institute, Cary, NC, United States). The 95% confidence interval for comparison was constructed using a *ch*i-squared likelihood ratio test (Table 2 and Supplementary Table S1) (McHugh, 2013). For multiple comparison analysis, ANOVA analysis and the Tukey-Kramer HSD *post hoc* multiple comparison was used in Tables 1, 3 (Tukey, 1949).

**TABLE 1:** Male percentage and mating success phenotypes of transgenic rescues, *him-4 (e1267)*, N2, β*pat-3(*+) and double mutants.

**Genotype**	**% Male (*n*)**	**Connecting letters report**	**Mating success (*n*)**
N2	0 (582)	. . C	+ (5)
pat-3(+)	0.34 (597)	. B C	+ (5)
*pat-3(Y792F)*	0.3 (897)	. B C	+ (40)
*pat-3(Y804F)*	0.9 (638)	. B C	+ (55)
*pat-3(Y792/804F)*	0.87 (575)	. B C	+ (14)
*pat-3(Y804E)*	4.3 (1179)	A . .	− (30)
*pat-3(*+)*; lon-2(e678) him-4(e1267)*	6.6 (437)	A . .	−(30)
*pat-3(Y792F); lon-2(e678) him-4(e1267)*	4.0 (682)	A B.	− (28)
*pat-3(Y804F); lon-2(e678) him-4(e1267)*	4.6 (480)	A . .	− (35)
*pat-3(Y792/804F); lon-2(e678) him-4(e1267)*	1.2 (1441)	. . C	+ (5)
*pat-3(Y792/804F); him-4(rh319)*	0.0 (2332)	. . C	ND
*lon-2(e678) him-4(e1267)*	4.7 (401)	A . .	− (15)
*him-4(rh319)*	6.9 (116)	A . .	ND

*The Connecting Letters Report shows results of an ANOVA test with Tukey-Kramer HSD *post hoc* multiple comparison. Strains not connected by the same letter are significantly different. Ordered differences report fo paired comparison, *p* > 0.05, is presented in Supplementary Table S2.*

**TABLE 2:** Male gonad migration and abnormal tail phenotype of transgenic rescues, *him-4 (e1267)*, N2, β*pat-3(*+) and double mutants.

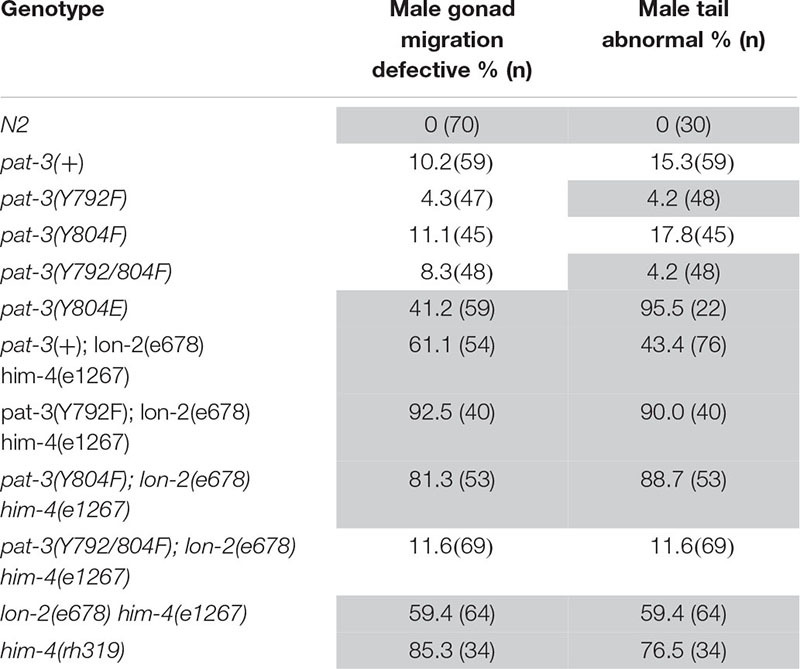

*Strains showing a significant difference from *pat-3(*+) for each phenotype, based on a 95% confidence interval for proportions, are shown in gray.*

**TABLE 3:** Inviable zygote phenotype of transgenic rescues, *him-4 (e1267)*, N2, β*pat-3(*+) and double mutants.

**Genotype**	**% Egg hatch (n)**	**Connecting letters report**
N2	88.0(750)	A. . .
*pat-3*(+)	88.8(507)	A. . .
*pat-3(Y792F)*	30.5(583)	. . C .
*pat-3(Y804F)*	40.1(524)	. B C .
*pat-3(Y792/804F)*	34.8(552)	. B C .
*pat-3(Y804E)*	40.2(602)	. B C .
*pat-3(*+*); lon-2(e678) him-4(e1267)*	42.4(1152)	. B . .
*pat-3(Y792F); lon-2(e678) him-4(e1267)*	12.5(522)	. . . D
*pat-3(Y804F); lon-2(e678) him-4(e1267)*	7.2(511)	. . . D
*pat-3(Y792/804F); lon-2(e678) him-4(e1267)*	96.6(1856)	A . . .
*pat-3(Y792/804F); him-4(rh319)*	47.8(2332)	. B . .
*lon-2(e678) him-4(e1267)*	30.0(1335)	. . C .
*him-4(rh319)*	9.06(116)	. . . D

*The Connecting Letters Report shows results of an ANOVA test with Tukey-Kramer HSD *post hoc* multiple comparison. Strains not connected by the same letter are significantly different. Ordered differences report for paired comparison, *p* > 0.05, is presented in Supplementary Table S3.*

## Results

### Tyrosine (Y) to Glutamic Acid (E) Mutation in the NPVY^804^ Motif Displayed Multiple Phenotypes Linked to the Mutation

**FIGURE 2:**
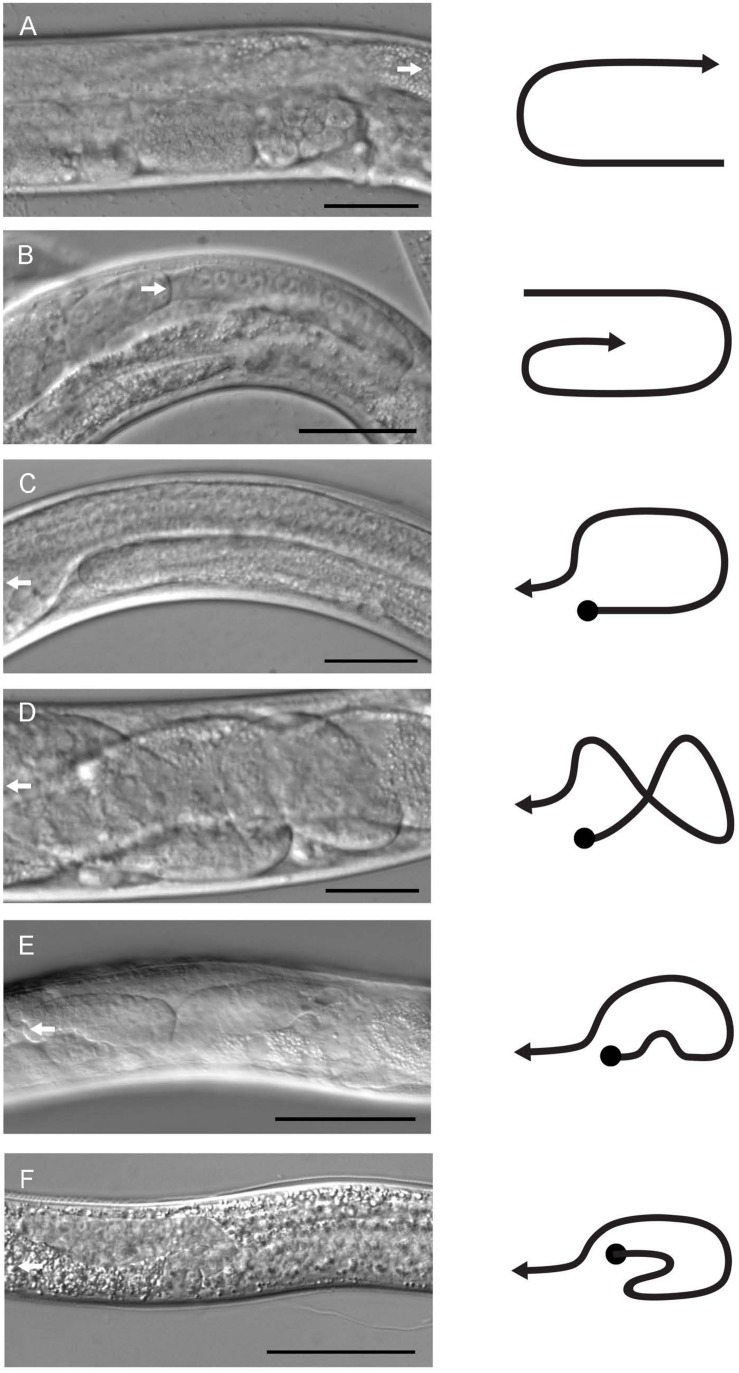
Gonad morphology of male and hermaphrodite mutant and transgenic animals. Gonad structures of control and *pat-3(Y804E)* animals were examined under DIC microscopy. **(A)** Control *pat-3(*+) hermaphrodite. **(B)** *pat-3(Y804E)* hermaphrodite. **(C)** *pat-3(*+) male. **(D,E)** *pat-3(Y804E)* male. **(F)** *him-4(e1267)* male. A white arrow indicates the direction of migrating leader cells, DTC for hermaphrodite **(A,B)** and linker cell for male **(C–E)**. Lines to the right of each image indicate gonad shapes. Bar = 50 μm.

**FIGURE 3:**
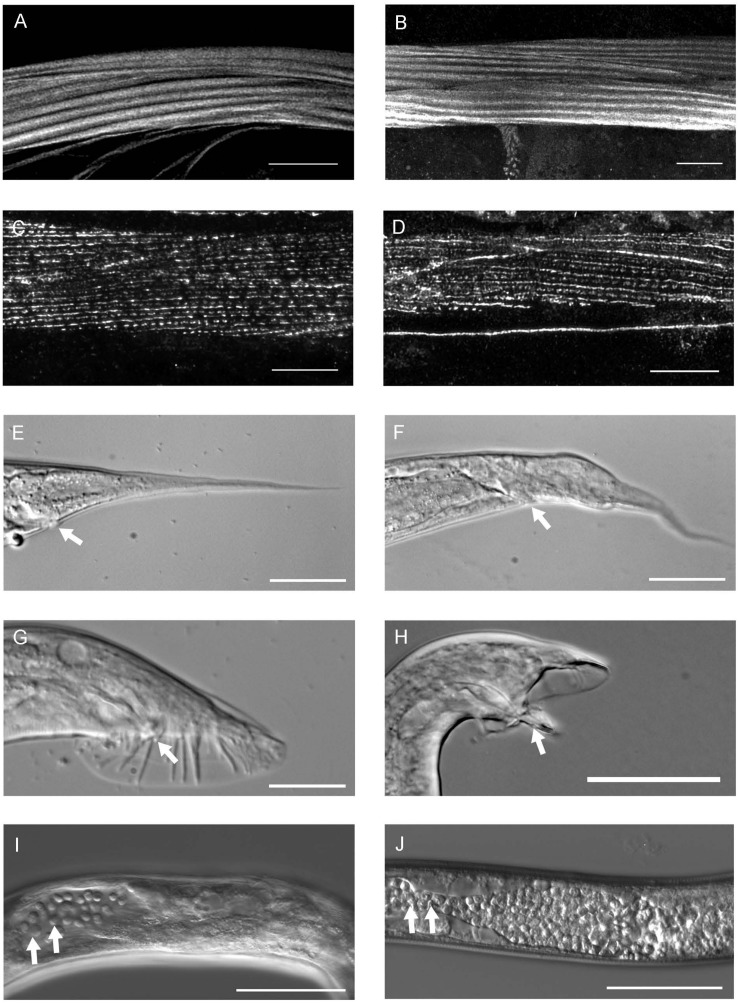
The phenotype of *pat-3(Y804E)* transgenic rescue animals. **(A)** *pat-3*(+) body wall muscle stained with rhodamine-phalloidin shows normal muscle filaments. **(B)** *pat-3(Y804E)* transgenic animal has organized muscle filament patterns; each filament is positioned in straight lines. **(C)** Monoclonal antibody MH25 stains *PAT-3* protein localized in the muscles of *pat-3*(+) and appeared with a regular distribution. **(D)** *pat-3(Y804E)* muscles showed regular dense bodies and M-line patterns. **(E)** *pat-3*(+) hermaphrodite tail. **(F)** The hermaphrodite tail has an abnormal shape and a protrusion in the base of the tail posterior to the anus (arrow). **(G)** *pat-3*(+) male tail with normal spicule indicated (arrow). **(H)** *pat-3(Y804E)* male tail appears abnormal with fused rays and protruding spicule (arrow). **(I)** *pat-3(Y804E)* gonad shows ectopic sperm (arrows) in the posterior body cavity near the end; in addition, panel **(J)**, *him-4(e1267)* gonad also shows a similar defect (arrows) to panel **(I)**. Bars = 50 μm.

In order to characterize the phospho-tyrosine motifs in the PAT-3 integrin cytoplasmic tail, we created a mutation that brings a negatively charged NPXY at the NPVY^804^ position. The Y to E mutagenesis was performed using *pat-3* genomic DNA as a template (Lee et al., 2001), and the completed *pat-3(Y804E)* construct was injected into a null allele, *pat-3(st564)*, to generate viable transgenic rescue lines with potential mutant phenotypes (Figure 1A). *kqEx804[pat-3 (Y804E)]*, the transgenic rescue animal, showed gonad migration defects. During gonad morphogenesis, two distal tips cells (DTC) at the polar ends of hermaphrodite gonad navigate the formation of the mirror image U-shaped tubular gonad (Lee et al., 2005; Xu et al., 2005). About 54.8% (*n* = 188) (Supplementary Table S1) of gonad arms in *pat-3(Y804E)* displayed abnormal turns and an irregular trajectory of distal arms (Figures 2A,B). Adult animals also appeared superficially wild type with slow movements, which led us to examine muscle organization. Actin cytoskeleton in the body wall muscle appeared normal; actin filaments were organized and regularly arranged (Figures 3A,B and Supplementary Figure S3). The distribution of PAT-3(Y804E) integrin was comparable to that of PAT-3(+), a wild type PAT-3 (Figures 3C,D). These data suggested that the mutant PAT-3 integrin is distributed in the body-wall muscle, resulting in a wild-type appearance, which parallels the phalloidin staining in Figure 3B. The transgenic rescue animals, however, showed an abnormal tail morphology. Unlike the normal appearance of the *pat-3(*+) wild-type rescue, *pat-3(Y804E)* animals displayed abnormal tails with irregular tapering at the end, 81.4% (*n* = 97) (Supplementary Table S1), of hermaphrodites (Figures 3E,F).

To investigate the effect of Y to E mutations in the membrane proxy NPXY motif, NPIY^792^ was also mutated to NPIE^792^; and a pPAT3(Y792E) genomic DNA construct was microinjected into *pat-3(st564)/dpy-19(e1259) glp-1(q339) III*, a balanced *pat-3* null (Figure 1A and see section “Materials and Methods”). However, the pPAT3(Y792E) construct failed to rescue the *pat-3* null allele. A transgenic line in wild-type background, *pat-3(Y792E, N2)*, was also created; it showed defects in tissues such as the hermaphrodite gonad, vulva, and tail caused from dominant effects of the *pat-3* mutant transgene (Supplementary Table S1).

### *pat-3(Y804E)* Displayed Him With Unsuccessful Male Mating and Abnormal Gonad

The *pat-3(Y804E)* transgenic recue animals displayed an unusually high number of males, 4.3% (*n* = 1179), resulting in a Him (high incidence of males) phenotype (Table 1). In *C. elegans*, the male percentage in the average population is as low as 0.2%. Mutants with the Him phenotype generally display higher occurrence of chromosome loss in mitotic germ cells, which increases the number of nullo-X ova or sperm in the gonad (Hodgkin, 1983). When the *pat-3(Y804E)* males were examined for mating (in 1 hermaphrodite to 3 to 6 males), we found no signs of mating success (Table 1 and see section “Materials and Methods”).

**FIGURE 4:**
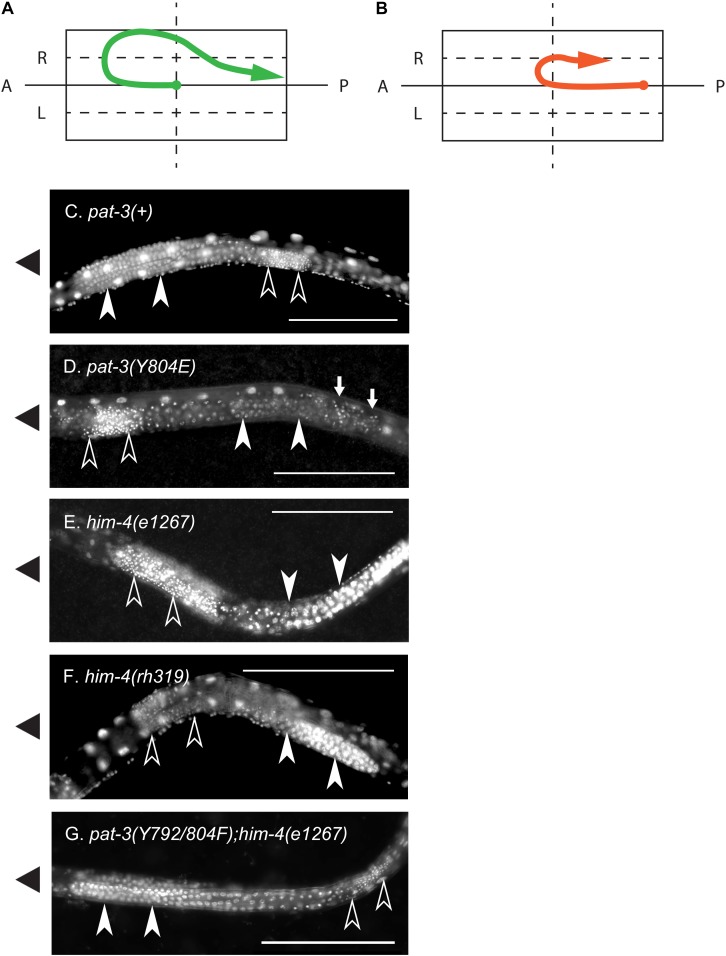
Nuclear staining of the defective gonad in *pat-3(Y804E)* and *him-4* mutant animals. **(A)** A linker cell migration trajectory of N2 male gonad. Male gonad initiates migration on the surface of ventral muscle (Hedgecock et al., 1987). The linker cell (arrow end) at the anterior tip of male gonad navigates the elongation of gonad tube, initiating its anterior migration at early L2 larval stage. Later it turns toward the dorsal muscle, starts to migrate posteriorly, slides down obliquely to the ventral side, and finally fuses to a rectal cell at the posterior end of the body. At the posterior end of the gonad, the distal tip cell (solid dot end) stays in the midbody. **(B)** A diagram of a *pat-3(Y804E)* or *him-4* mutant gonad. The mutant gonad displays abnormal turns. The linker cell (arrow) fails to make dorsal turns and loops around the ventral half instead. In many cases, distal tip (solid dot) appears extended toward the posterior end of the body. **(C)** Nuclear staining of *pat-3(*+) male, showing a typical male gonad appearance. From the anterior, the turning gonad arm is filled with undifferentiated germ cell nuclei. **(D)** Nuclear staining of *pat-3(Y804E)*, unlike the *pat-3(*+) male gonad in panel **(C)**, this gonad displayed sperm nuclei located anteriorly to undifferentiated germ cell nuclei. Small arrows indicate ectopic sperm. **(E,F)** Alleles of *him-4* mutants; nuclear staining patterns are similar to that of *pat-3(Y804E)*. **(G)** *pat-3(Y792/804F); him-4(e1267) lon-2(e678)*, showing the gonad with rescued morphology. This gonad displayed sperm nuclei located posterior to undifferentiated germ cells. Ventral side was determined by the location of male tail fan opening and ventral nerve cord staining. Sperm nuclei (small puncta, open arrow heads) are located posterior to undifferentiated germ cell nuclei (closed arrow heads). Worms were fixed with methanol and stained with 0.1 μg/ml DAPI in M9 buffer (see section “Materials and Methods”). The solid triangles on the left indicate the anterior end of animals. Bar = 100 μm.

In *C. elegans*, males have a distinct gonad and copulatory structure specialized for sperm production and mating (Emmons, 2005). The male gonad has an asymmetric appearance formed by J-shaped migration of the linker cell at the proximal tip while the distal tip of gonad becomes static at the ventral midbody (Figures 2C, 4A) (Antebi et al., 1997; Emmons, 2005). The male tail, a genital structure composed of spicules, rays, and a fan (Klass et al., 1976), is connected to a vas deferens at the linker cell end of the gonad.

The unsuccessful mating of *pat-3(Y804E)* males led us to investigate the cause of the mating defect, which can be caused by failed gonadogenesis (Emmons, 2005) or abnormal male genitals (Chow et al., 1995). The *pat-3(Y804E)* male gonad was examined under DIC microscopy. Distal gonad migration appeared abnormal; the migration path of the cell failed to show a typical pattern. Instead, the gonad tube appeared to make irregular turns (Figures 2D,E, 4B). The gonad often appeared displaced; the distal tip was anchored in various abnormal positions, along with defects such as extra loops around the ventral plane anterior or posterior to the vulva (41.2%, *n* = 59) (Table 1). In addition, *pat-3(Y804E)* male tails showed abnormal rays and spicules (Figures 3G,H); most failed to have a regular ray arrangement, instead of having deformed fans and protruded spicules (Figure 3H). It should be noted that there was a low percentage of gonad migration and tail morphology defects in *pat-3(*+) animals (Table 2). Next, *pat-3(Y804E)* males were also stained with DAPI to examine the nuclear morphology of the male gonad. The majority of males showed a typical gonad appearance but some showed ectopic puncta; staining of ectopic sperm nuclei was dispersed in the body cavity.

In contrast to germ cells being located anterior to sperm in *pat-3(*+) (Figure 4C), *pat-3(Y804E)* male gonads displayed undifferentiated germ cells located posterior to sperm (Figures 3I, 4D). Briefly, the gonad linker cell migrates and extends the proximal gonad arm posteriorly to fuse to a rectal cell (Sulston et al., 1980; Friedman et al., 2000; Vogel and Hedgecock, 2001; Abraham et al., 2007). Therefore, *pat-3(*+) or N2 sperm nuclei normally appear posterior to the undifferentiated germ cell nuclei in nuclear staining (Figure 4C). The *pat-3(Y804E)* male gonad, however, displayed unique germ cell positioning, suggesting that the proximal end of the gonad failed its posterior migration or failed to fuse firmly to the rectum.

### Defects in *pat-3(Y804E)* Are Analogous to the Phenotypes of *him-4/hemicentin* Alleles

The phenotypes of the *pat-3(Y804E)* transgenic line suggested that *pat-3(Y804E)* defects could be analogous to the phenotype of *him-4* alleles (Hodgkin et al., 1979). The *him-4* gene encodes for hemicentin, a large ECM protein with a von Willebrand A domain, immunoglobulin repeats, and epidermal growth factor domains, similar to human hemicentin I (Vogel and Hedgecock, 2001; Schultz et al., 2005; Dong et al., 2006; Segade, 2010; Feitosa et al., 2012); *him-4* alleles shared Him and unsuccessful mating phenotypic descriptions with *pat-3(Y804E)* (Table 1). The *him-4(e1267)* mutant animals displayed Him, unsuccessful mating, and tail abnormality comparable to that of *pat-3(Y804E)* (Tables 1, 2). The mutant *him-4* also showed a displaced distal gonad (Figure 2F), ectopic sperm (Figure 3J), and an abnormal arrangement of sperm and undifferentiated germ cells (Figure 4E). We also examined the male gonad of *him-4(rh319)*, a *him-4* null allele (Vogel and Hedgecock, 2001); *rh319* showed similar phenotypes comparable to *e1267* in the appearance (85.3%, *n* = 34; Table 2) of the ectopic sperm (Figure 4F). Both *him-4(e1267)* and *pat-3(Y804E)*, however, showed muscle filament appearance comparable to that of *pat-3(*+) animals (Supplementary Figure S2). The similar phenotypes between the *him-4* mutant alleles and *pat-3(Y804E)* suggested that *pat-3* is linked to *him-4* in gonad formation and function.

### The Role of *him-4* in *pat-3* NPXY Modulation

The phenotypic similarity between *him-4* alleles and *pat-3(Y804E)* suggested that the wild-type *him-4* affects tyrosine (Y) at the NPVY^804^ motif to modulate integrin functions. In order to compare the role of NPXY mutations, we also reexamined the phenotype in non-polar NPXY mutants, *pat-3(Y792F)*, *pat-3(Y804F)*, and *pat-3(Y792/804F)* transgenic rescue animals from our previous analyses (Lee et al., 2001; Xu et al., 2010; Tables 1–3). In all Y to F mutants, the abnormal male gonad migration was observed in about 10% or fewer, which is comparable to *pat-3(*+). Male tail morphology appeared wild-type in *pat-3(Y792F)* and *pat-3(Y792/804F)*, comparable to that of N2. The *pat-3(Y804F)* animals, however, showed minor defects in tail morphology (Table 2). Despite slight variations in male gonad and tail defect (Table 2), however, their males retained the ability to mate (Table 1). In addition to minor defects, the three Y to F mutants displayed low hatching rates due to inviable zygotes (Table 3), although none of these mutants displayed Him or ectopic sperm similar to *pat-3(Y804E)*.

In order to test the role of *him-4* in NPXY modulation, the double mutants, *pat-3(Y792F); him-4(e1267)*, *pat-3(Y804F); him-4(e1267)*, and *pat-3(Y792/804F); him-4(e1267)* were generated. Our analysis showed that the male percentage in *pat-3(Y792F); him-4(e1267)* and *pat-3(Y804F); him-4(e1267)* were comparable to *him-4(e1267)* (Table 1), while that of *pat-3(Y792/804F); him-4(e1267)* was much lower than *him-4(e1267)*. Similar analysis was performed with the *him-4(rh319)* null mutant; the male percentage of *pat-3(Y792/804F); him-4(rh319)* double showed no signs of male. This suggested that *pat-3(Y792/804F)* suppresses the Him phenotype of *him-4(e1267)* and *him-4(rh319)* to a significantly low level (Table 1).

Next, *pat-3(Y792F); him-4(e1267)* and *pat-3(Y804F); him-4(e1267)* showed increased abnormal gonad (Supplementary Figure S1); animals showed ectopic sperm and gonad migration phenotypes, 92.5% (*n* = 40) and 81.3% (*n* = 53), respectively, which was significantly higher level than that of the *him-4* alleles (*chi*-squared likelihood ratio *p* < 0.0002, Table 2, and Supplementary Figure S1). In contrast, *pat-3(Y792/804F); him-4(e1267)* animals showed the defects in 11.6% (*n* = 69), which was lower than that of the *him-4* allele (Figure 4G and Supplementary Figure S1) (59.4%, *n* = 64). Male tail morphology was also examined (Table 2). Two double mutants, *pat-3(Y792F); him-4(e1267)* and *pat-3(Y804F); him-4(e1267)*, showed significantly increased in abnormal male tails with deformed genitals such as protruded spicules and irregular fans, 90% (*n* = 40) and 88.7% (*n* = 53), respectively (*chi*-squared likelihood ratio *p*-value of <0.0001, Table 2). In contrast, the tail abnormal of *pat-3(Y792/804F); him-4(e1267)* animals appeared as low as 11.6% (*n* = 69), suggesting that *pat-3(Y792/804F)* suppressed the tail defects of *him-4*. We also measured male mating; analyses of the double mutants revealed that *pat-3(Y792/804F); him-4(e1267)* were able to suppress *him-4* male mating phenotype. Only *pat-3(Y792/804F); him-4(e1267)* males among other double mutants were able to mate with hermaphrodites and produce progeny from mating (Table 1).

In addition to mating failure, *him-4* mutants had many eggs failed to hatch (inviable zygote) due to aneuploidy of autosomal chromosomes (Hodgkin, 1983). The *him-4(e1267)* and *him-4(rh319)* animals displayed 30% (*n* = 1335) and 9.6% (*n* = 116) egg hatching, respectively. The *pat-3(Y792F), pat-3(Y804F), pat-3(Y804E)*, and *pat-3(Y792/804F)* animals were also comparable to the hatching rate of *him-4(e1267)* mutant animals (Table 3). We then measured the hatching rate in the double mutants, *pat-3(Y792F); him-4(e1267)*, *pat-3(Y804F); him-4(e1267)*, *pat-3(Y792/804F); him-4(e1267)*, and *pat-3(Y792/804F); him-4(rh319)*. Introduction of *him-4* into *pat-3* Y to F single mutants exacerbated hatching defects (Table 3). However, the hatching rate of *pat-3(Y792/804F); him-4(e1267)* eggs was comparable to that of N2 and *pat-3(*+). The hatching of *pat-3(Y792/804F); him-4(rh319) was significantly increased compared to the rate of him-4(rh319)* (Table 3), suggesting that *pat-3* non-polar mutation in both NPXY also suppresses the inviable zygote phenotype of *him-4 mutations*.

## Discussion

In this study, the function of the distal NPVY^804^ motif of β*pat-3* integrin cytoplasmic tail was analyzed. The Y to E mutation inducing negatively charged NPXY introduced to the distal motif fails to affect the muscle organization but causes defects in the gonad and tail. This mutant *pat-3* integrin also shows a Him phenotype undescribed in previous integrin studies. The phenotypes of *pat-3(Y804E)* animals resemble defects in *him-4* alleles. Double mutant analysis reveals that non-polar *pat-3* mutants, *pat-3(Y792/804F)*, suppresses *him-4* phenotypes, whereas *pat-3(Y792F)* or *pat-3(Y804F)* enhances the gonad phenotype of *him-4*, suggesting that HIM-4/hemicentin interacts with *pat-3* integrin and prevents the chemical modification of both NPXY motifs in the cytoplasmic tail of PAT-3.

### The Phenotypes of *pat-3(Y804E)* Are Analogous to That of *him-4* Mutation

The Him phenotype generally results from aneuploidy of mitotic germ cells (Hodgkin et al., 1979). In *him-4* mutants, the Him phenotype is a result of aberrant cellularization in mitotic germ cells, which mainly occurs due to incomplete constriction of the rachis (Vogel and Hedgecock, 2001; Dong et al., 2006). It also suggested that the failed cytokinesis is strongly linked to the low hatching rate of fertilized eggs in *him-4*. It appears that the Him phenotype is only linked to *pat-3(Y804E)*, although two Y to F mutants, *pat-3(Y804F)* and *pat-3(Y792/804F)*, appear to have a higher male number than *pat-3(*+). Our ANOVA testing and a *post hoc* multiple comparison indicates that the male percentage of *pat-3(*+), *pat-3(Y792F)*, *pat-3(Y804F)*, and *pat-3(Y792/804F)* are not significantly different from each other (Table 1).

Male gonad defects also appear specific to the *pat-3(Y804E)* mutation (Table 2). The male of *pat-3(Y804E)* or *him-4* contains ectopic sperm in the vas deferens or in the body cavity, which is undescribed in previous *pat-3* integrin studies (Lee et al., 2001, 2005; Xu et al., 2005, 2010; Kihira et al., 2012). The *mab-23* and *egl-38* mutants show a Mab (male abnormal) phenotype that is similar to the gonad defects of *pat-3(Y804E)* or *him-4* mutant. In these mutants, the gonad linker cell fails to die and be engulfed by U lineage (*U.lp* and *U.rp*) epithelial cells to make an opening to the cloaca (Sulston et al., 1980; Abraham et al., 2007). Consequently, sperm accumulate in the vas deferens or the body cavity (Lints and Emmons, 2002), suggesting that this failure prevents insemination and is the leading cause of male sterility. There are low percentage gonad defects in *pat-3(*+) along with other Y to F mutant males (Table 2), but the Y to F males with these minor defects fail to show male sterility (Table 1).

HIM-4/hemicentin is localized in between the two BM from adjacent tissues and forms puncta patterns at the cell anchoring points (Morrissey et al., 2014). The Y804E mutation shows a high percentage of abnormal male tails, possibly resulting from the instability of BM-BM attachment between muscle and tail hypodermis. The *pat-3(*+) and Y to F mutant males display a low percentage of tail defects. However, these males with a tail defect fertilize hermaphrodites and produce progeny (Table 1).

We suggest that HIM-4/hemicentin forms complex with other ECM proteins such as collagen type IV, laminins, perlecan, or other fibulins (Kramer, 2005) and these complexes interact with integrin molecules. There are several reports on the interaction of integrins with homologous fibulins in mammalian cells (Timpl et al., 2003). The studies of *him-4*/hemicentin in anchor cell invasion have revealed that *him-4* is colocalized with β*pat-3* integrin and *vab-10/plakin* at the attachment structure of hypodermal seam and uterine seam cells (Morrissey et al., 2014). However, biochemical details of the direct or indirect interactions between *him-4/hemicentin* and βPAT-3 integrin are yet to be addressed and may be the subject of future studies.

### HIM-4/Hemicentin Likely Links to NPXY Modification in β*pat-3* Integrin

The double mutant analyses indicate that non-polar integrin, *pat-3(Y792/804F)*, significantly suppresses Him and mating defects, gonad morphology defects, and embryonic lethal phenotypes of the *him-4* allele (Tables 1–3). We would like to propose that the phenotypes of the *him-4* loss-of-function allele might be caused by negatively charged NPXY motif and that the wild-type function of *him-4* likely maintains NPXY motifs uncharged. The severe abnormal gonad and increased inviable zygote phenotypes in *pat-3(Y792F); him-4(e1267)* and *pat-3(Y804F); him-4(e1267)* males (Tables 1, 3) are potentially due to increased negative charge or phosphorylation on the counter NPXY motif of PAT-3 molecules. For example, *pat-3(Y792F); him-4(e1267)* may increase negatively charged NPVY^804^ locally, while there is the similar increase of negatively charged NPIY^792^ in *pat-3(Y804F); him-4(e1267)*. The *him-4(e1267)* and *him-4(rh319)* phenotypes are significantly suppressed in *pat-3(Y792/804F); him-4(e1267) and him-4(rh319)* double mutants. In this case, both NPXY motifs are kept from the modification, which is similar to default condition, uncharged NPXY motifs.

Our new findings provide valuable information about the function of NPXY motifs in β integrin. They provide us with new insights into how cell-matrix interactions modulate the conserved phosphorylation motifs in the integrin tail. This information will help us to understand integrin and hemicentin functions in many species. Finally, because integrin has been implicated in human pathology such as tumor growth, inflammation, and other diseases, this foundational research on the interaction between β integrin and hemicentin may lead toward gains in research related to human health.

## Data Availability Statement

The datasets generated for this study are available on request to the corresponding author.

## Author Contributions

ZQ, PS, JA, and E-JY performed the experiments. PS performed the statistical analyses. ZQ and PS wrote experimental procedures and figure legends, and generated figures and Supplementary Materials. ML wrote main manuscript text.

## Conflict of Interest

The authors declare that the research was conducted in the absence of any commercial or financial relationships that could be construed as a potential conflict of interest.
