# Supplementary material for: A Novel Mutation in an NPXY Motif of β Integrin Reveals Phenotypes Similar to him-4/hemicentin
Source: Front Cell Dev Biol. 2019 Oct 28;7:247. doi: 10.3389/fcell.2019.00247 (PMC6827421; doi:10.3389/fcell.2019.00247)

**Supplementary Data**

**Table S1. Phenotype comparison of *pat-3* transgenic hermaphrodite**

| **Transgenic lines** |  | **% DTC Mig (n)** |  | **% Protruded vulva (n)** |  | **% Tail Defect (n)** |
| --- | --- | --- | --- | --- | --- | --- |
| *kqEx792(Y792E; N2)* |  | 79.9 (289) |  | 80.2 (131) |  | 20.4 (142) |
| *kqEx51(Y804E; N2)* |  | 49.1 (161) |  | 9.2 (87) |  | 30.1 (83) |
| *kqEx804(Y804E)* |  | 54.8 (188) |  | 71.1 (97) |  | 81.4 (97) |
| *mwEx443[pat-3(+)]* |  | 18 (90) |  | 0.9 (45) |  | 0.9 (45) |

Strains showing a significant difference from *mwEx443 pat-3(+)* for each phenotype, based on a 95% confidence interval for proportions, are shown in gray.

Table S2. Ordered Differences Report of paired comparison, Him phenotype data (*p > .05*) in the Table 1.

| *Level* | *- Level* | Difference | Std Err Dif | Lower CL | Upper CL | p-Value |
| --- | --- | --- | --- | --- | --- | --- |
| *Y792F; e1267* | *Y792F* | 3.7306 | 1.138399 | -0.17982 | 7.64102 | 0.0758 |
| *Y792F; e1267* | *pat-3(+)* | 3.64125 | 1.199978 | -0.48069 | 7.76319 | 0.1367 |
| *Y792F; e1267* | *YYFF* | 3.533143 | 1.063667 | -0.12057 | 7.18685 | 0.0676 |
| *Y792F; e1267* | *Y804F* | 3.0768 | 1.138399 | -0.83362 | 6.98722 | 0.2743 |
| *r319* | *Y792F; e1267* | 2.874 | 1.138399 | -1.03642 | 6.78442 | 0.3761 |
| *pat-3(+); e1267* | *Y792F; e1267* | 2.703875 | 1.039211 | -0.86583 | 6.27358 | 0.3298 |
| *r319* | *Y804E* | 2.458143 | 0.993676 | -0.95515 | 5.87143 | 0.4082 |
| *r319* | *Y804F; e1267* | 2.424 | 0.967453 | -0.89921 | 5.74721 | 0.388 |
| *pat-3(+); e1267* | *Y804E* | 2.288018 | 0.878294 | -0.72893 | 5.30497 | 0.3279 |
| *r319* | *e1267* | 2.2594 | 1.073293 | -1.42737 | 5.94617 | 0.66 |
| *pat-3(+); e1267* | *Y804F; e1267* | 2.253875 | 0.848513 | -0.66078 | 5.16853 | 0.2991 |
| *pat-3(+); e1267* | *e1267* | 2.089275 | 0.967453 | -1.23394 | 5.41249 | 0.6227 |
| *Y804F* | *N2* | 0.9322 | 1.073293 | -2.75457 | 4.61897 | 0.9996 |
| *Y804F* | *YYFF; r319* | 0.9322 | 0.915307 | -2.21189 | 4.07629 | 0.9981 |
| *Y804F* | *YYFF; e1267* | 0.9322 | 1.0276 | -2.59762 | 4.46202 | 0.9994 |
| *Y804F* | *Y792F* | 0.6538 | 1.073293 | -3.03297 | 4.34057 | 1 |
| *e1267* | *Y792F; e1267* | 0.6146 | 1.138399 | -3.29582 | 4.52502 | 1 |
| *Y804F* | *pat-3(+)* | 0.56445 | 1.138399 | -3.34597 | 4.47487 | 1 |
| *YYFF* | *N2* | 0.475857 | 0.993676 | -2.93743 | 3.88915 | 1 |
| *YYFF* | *YYFF; r319* | 0.475857 | 0.820501 | -2.34257 | 3.29429 | 1 |
| *YYFF* | *YYFF; e1267* | 0.475857 | 0.944138 | -2.76727 | 3.71898 | 1 |
| *Y804F* | *YYFF* | 0.456343 | 0.993676 | -2.95695 | 3.86963 | 1 |
| *Y804F; e1267* | *Y792F; e1267* | 0.45 | 1.039211 | -3.1197 | 4.0197 | 1 |
| *Y804E* | *Y792F; e1267* | 0.415857 | 1.063667 | -3.23785 | 4.06957 | 1 |
| *pat-3(+)* | *N2* | 0.36775 | 1.138399 | -3.54267 | 4.27817 | 1 |
| *pat-3(+)* | *YYFF; r319* | 0.36775 | 0.990849 | -3.03583 | 3.77133 | 1 |
| *pat-3(+)* | *YYFF; e1267* | 0.36775 | 1.095425 | -3.39505 | 4.13055 | 1 |
| *Y792F* | *N2* | 0.2784 | 1.073293 | -3.40837 | 3.96517 | 1 |
| *Y792F* | *YYFF; r319* | 0.2784 | 0.915307 | -2.86569 | 3.42249 | 1 |
| *Y792F* | *YYFF; e1267* | 0.2784 | 1.0276 | -3.25142 | 3.80822 | 1 |
| *e1267* | *Y804E* | 0.198743 | 0.993676 | -3.21455 | 3.61203 | 1 |
| *YYFF* | *Y792F* | 0.197457 | 0.993676 | -3.21583 | 3.61075 | 1 |
| *r319* | *pat-3(+); e1267* | 0.170125 | 0.967453 | -3.15309 | 3.49334 | 1 |
| *e1267* | *Y804F; e1267* | 0.1646 | 0.967453 | -3.15861 | 3.48781 | 1 |
| *YYFF* | *pat-3(+)* | 0.108107 | 1.063667 | -3.5456 | 3.76182 | 1 |
| *pat-3(+)* | *Y792F* | 0.08935 | 1.138399 | -3.82107 | 3.99977 | 1 |
| *Y804F; e1267* | *Y804E* | 0.034143 | 0.878294 | -2.98281 | 3.05109 | 1 |
| *YYFF; r319* | *N2* | 0 | 0.915307 | -3.14409 | 3.14409 | 1 |
| *YYFF; e1267* | *N2* | 0 | 1.0276 | -3.52982 | 3.52982 | 1 |
| *YYFF; e1267* | *YYFF; r319* | 0 | 0.861273 | -2.95848 | 2.95848 | 1 |

Table S3. Ordered Differences Report paired comparison, hatching percentage data (*p > .05*) in Table 3.

| Level | - Level | Difference | Std Err Dif | Lower CL | Upper CL | p-Value |
| --- | --- | --- | --- | --- | --- | --- |
| *YYFF; r319* | *YYFF* | 11.55273 | 3.613907 | -1.0524 | 24.1578 | 0.1018 |
| *Y804E* | *Y792F* | 9.45333 | 4.530273 | -6.348 | 25.2547 | 0.6719 |
| *pat-3(+); e1267* | *YYFF* | 9.285 | 3.923331 | -4.3994 | 22.9694 | 0.4836 |
| *Y804E* | *e1267* | 9.282 | 4.051999 | -4.8512 | 23.4152 | 0.5345 |
| *Y804F* | *Y792F* | 9.23667 | 4.530273 | -6.5647 | 25.038 | 0.703 |
| *Y804F* | *e1267* | 9.06533 | 4.051999 | -5.0678 | 23.1985 | 0.5707 |
| *YYFF; e1267* | *pat-3(+)* | 7.855 | 3.923331 | -5.8294 | 21.5394 | 0.7262 |
| *YYFF; r319* | *Y804F* | 7.67939 | 3.613907 | -4.9257 | 20.2845 | 0.6466 |
| *YYFF; r319* | *Y804E* | 7.46273 | 3.613907 | -5.1424 | 20.0678 | 0.6861 |
| *pat-3(+); e1267* | *Y804F* | 5.41167 | 3.923331 | -8.2727 | 19.096 | 0.9724 |
| *YYFF* | *Y792F* | 5.36333 | 4.530273 | -10.438 | 21.1647 | 0.992 |
| *Y792F; e1267* | *Y804F; e1267* | 5.22 | 4.530273 | -10.5813 | 21.0213 | 0.9937 |
| *pat-3(+); e1267* | *Y804E* | 5.195 | 3.923331 | -8.4894 | 18.8794 | 0.9799 |
| *YYFF* | *e1267* | 5.192 | 4.051999 | -8.9412 | 19.3252 | 0.9845 |
| *YYFF; e1267* | *N2* | 4.44167 | 3.203386 | -6.7316 | 15.6149 | 0.9712 |
| *Y804E* | *YYFF* | 4.09 | 4.530273 | -11.7113 | 19.8913 | 0.9994 |
| *Y804F* | *YYFF* | 3.87333 | 4.530273 | -11.928 | 19.6747 | 0.9996 |
| *N2* | *pat-3(+)* | 3.41333 | 3.923331 | -10.271 | 17.0977 | 0.9996 |
| *Y792F; e1267* | *r319* | 2.70067 | 4.051999 | -11.4325 | 16.8338 | 1 |
| *r319* | *Y804F; e1267* | 2.51933 | 4.051999 | -11.6138 | 16.6525 | 1 |
| *YYFF; r319* | *pat-3(+); e1267* | 2.26773 | 2.815934 | -7.5541 | 12.0896 | 0.9998 |
| *Y804E* | *Y804F* | 0.21667 | 4.530273 | -15.5847 | 16.018 | 1 |
| *e1267* | *Y792F* | 0.17133 | 4.051999 | -13.9618 | 14.3045 | 1 |

Figure S1. Nuclear staining of double mutants

Nuclear staining of *pat-3; him-4* mutants was compared to that of pat-3(+). Panel A. N2; panel B. *pat-3(Y792F); him-4(e1267) lon-2(e678)*; panel C. *pat-3(Y804F); him-4(e1267) lon-2(e678)*; panel D. *pat-3(Y792/804F); him-4(e1267) lon-2(e678),* showing gonad with rescued morphology. Worms were stained with 0.1 μg/ml DAPI (see Materials and Methods). Each panel represents a worm with defective or rescued gonad. Open arrowheads indicate sperm nuclei; closed arrowheads indicate undifferentiated germ cells. Bars = 100 μm.

Figure S2. Fluorescent Staining of wild-type N2, *pat-3(+)* transgenic worm rescued with wild-type *pat-3* gene, *pat-3(Y804E)*, and *him-4(e1267)* animals. Panels A and B, monoclonal antibody MH25 stains PAT-3 protein localized in the muscles of N2 (A) and *pat-3(+)* (B). Both appeared with a regular distribution. Panels C and D, *pat-3(Y804E)* and *him-4(e1267),* respectively, muscles showed regular dense bodies and M-line patterns. Panels E and F, *raIs8[UNC-112::GFP, rol-6(su1006)]* animals were treated with control (panel E) and *him-4* RNAi bacteria (panel F). Bar = 10 µm.

Figure S3. Muscle Tissue with Phalloidin Fluorescent Staining. Panels A and B, N2 and *pat-3(+),* body wall muscle stained with rhodamine-phalloidin shows normal muscle filaments. Panels C and D, *pat-3(Y804E)* transgenic animal (C) and *him-4(e1267) (D)*, display organized muscle filament patterns; each filament is positioned in straight lines. Bar = 10 µm.


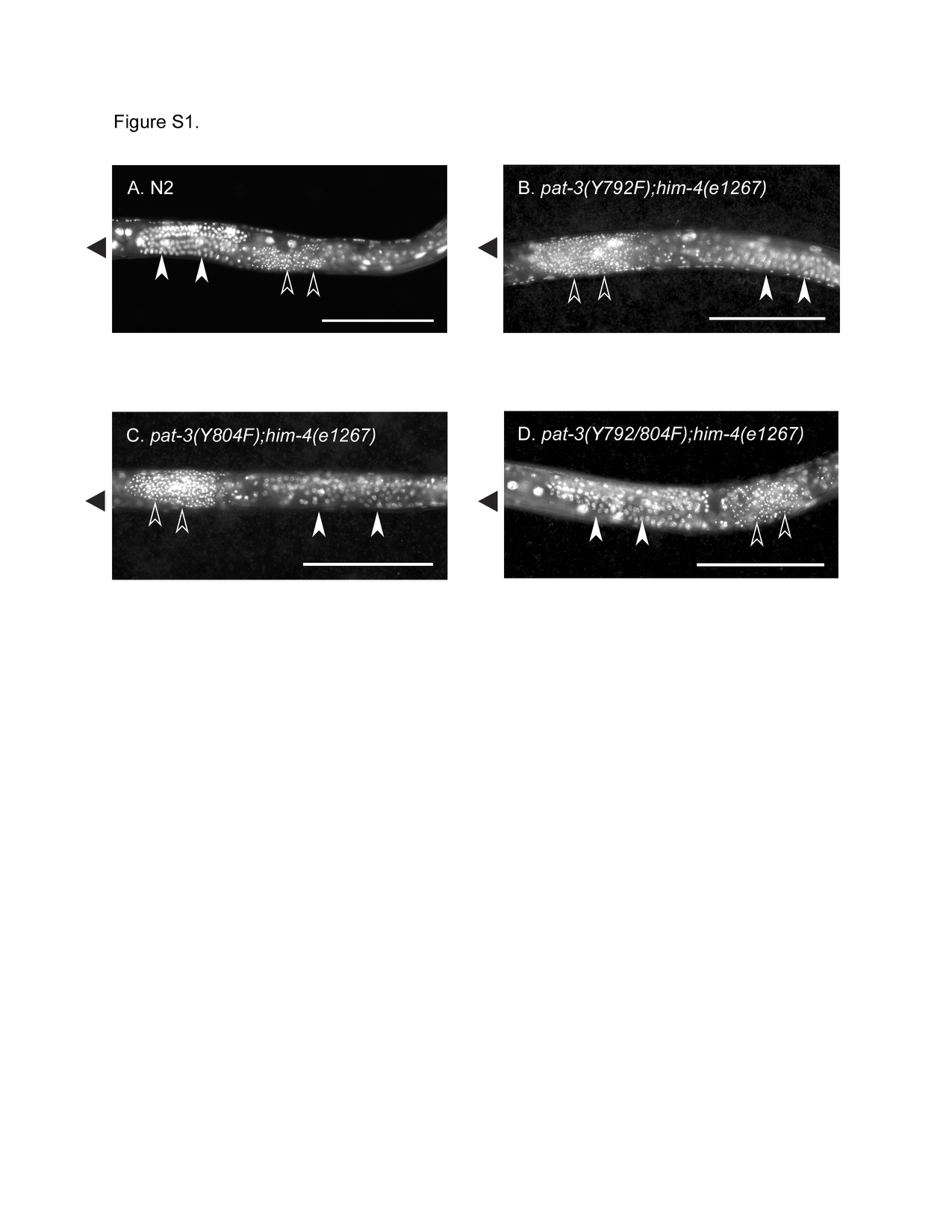


Figure S2.


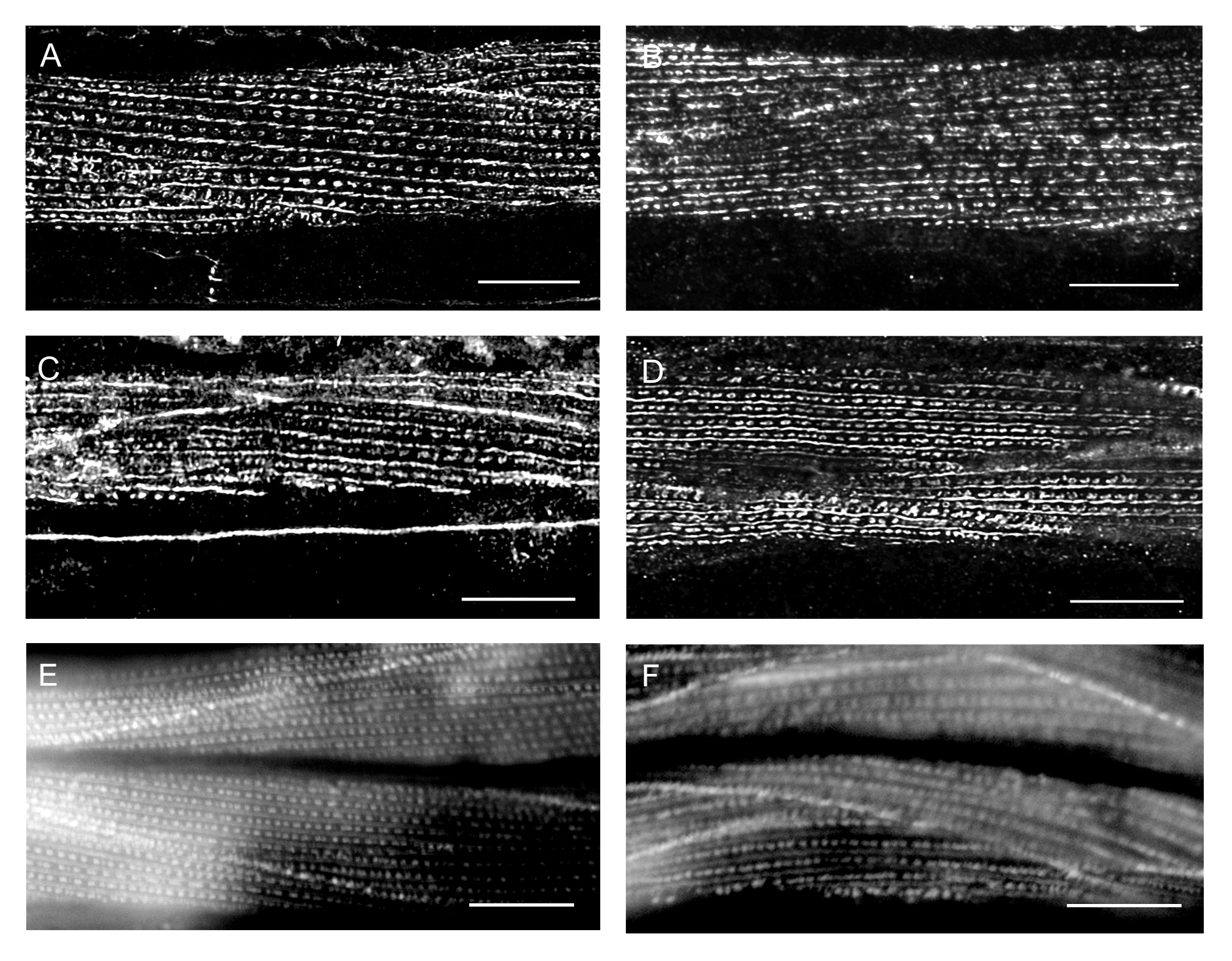


Figure S3.


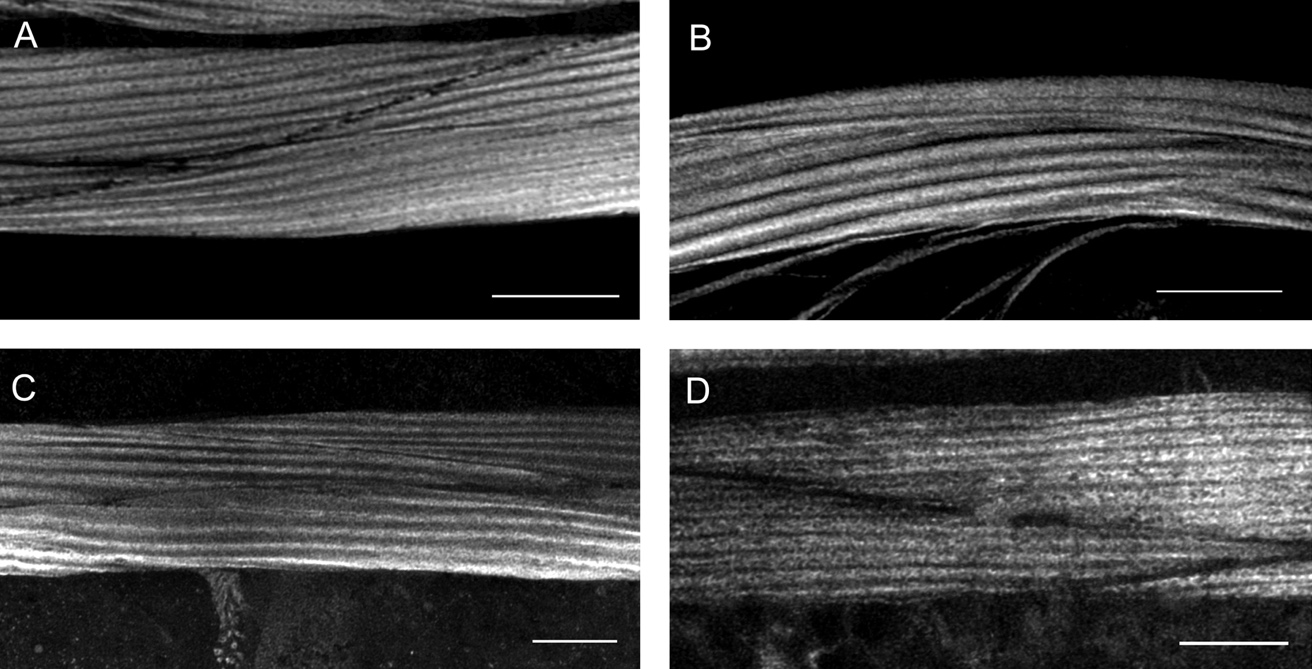

Supplement: Supplementary file 1 [file Data_Sheet_1.docx]
